# A Novel Tetravalent Bispecific Immune Cell Engager Activates Natural Killer Cells to Kill Cancer Cells without Mediating Fratricide

**DOI:** 10.3390/antib13030075

**Published:** 2024-09-10

**Authors:** Ge Yang, Shahryar Khoshtinat Nikkhoi, Hajar Owji, Geng Li, Mohammad Massumi, Jessica Cervelli, Venu Gopal Vandavasi, Arash Hatefi

**Affiliations:** 1 Department of Pharmaceutics, Rutgers University, Piscataway, NJ 08854, USA; 2 Environmental and Occupational Health Science Institute, Flow Cytometry Core Facility, Rutgers University, Piscataway, NJ 08854, USA; 3 Department of Chemistry, Biophysics Core Facility, Princeton University, Princeton, NJ 08544, USA; 4 Cancer Pharmacology Program, Cancer Institute of New Jersey, New Brunswick, NJ 08901, USA

**Keywords:** multi-paratope antibody, BiKE, immune cell engager, VHH, tetravalent bispecific antibody, fratricide, daratumumab, CD16a, laNK92

## Abstract

We previously reported the structure, affinity, and anticancer activity of a bivalent bispecific natural killer cell engager (BiKE) composed of one anti-CD16a VHH and one anti-HER2 VHH fused via a linker. In this study, we explored the engineering of a tetravalent BiKE by fusing two anti-CD16a and two anti-HER2 VHHs in tandem, using bivalent BiKE as a template. The tetravalent BiKE was genetically engineered, and its tertiary structure was predicted using in silico modeling. The antigen binding and affinity of the tetravalent BiKE were assessed using ELISA, flow cytometry, and biolayer interferometry. The ability of the BiKEs to kill cancer cells was evaluated through classical and residual antibody-dependent cellular cytotoxicity (ADCC) assays. Additionally, we investigated the potential for NK cell fratricide via CD16a-CD16a crosslinking. Our results revealed that the tetravalent BiKE exhibited at least 100-fold higher affinity toward its target antigens compared to its bivalent counterpart. The residual ADCC assay indicated that the tetravalent BiKE was more effective in killing cancer cells than the bivalent BiKE, attributable to its lower K_off_ value, which prolonged its binding to NK cell surfaces. Fratricide assays demonstrated that neither the bivalent nor the tetravalent BiKE mediated fratricide. Notably, our findings showed that daratumumab-induced NK fratricide was restricted to CD38-CD38 crosslinking and was not related to ADCC via CD16a-CD38 crosslinking. This study is the first in the literature to show the successful engineering of a tetravalent immune cell engager composed of tandem VHH units, which achieves high affinity and anticancer activity without mediating fratricide.

## 1. Introduction

Variable domains of heavy chain-only antibodies (VHH) are single-domain antibodies derived from camelid heavy chain-only antibodies [1]. VHHs were first discovered in 1993 and are commonly referred to as nanobodies, a term originally coined by the company Ablynx in 2003 [2]. The monomeric, highly structured scaffold of VHHs imparts antigen binding abilities comparable to conventional monoclonal antibodies (mAbs) but with ten times smaller size and greater thermostability. These characteristics make VHHs suitable for the modular design of multivalent antibody drugs. The small size and stable architecture of the VHHs facilitate improved avidity through the creation of bi-, tri, or multi-paratope forms. Multivalent VHHs, composed of repeating units of the same VHH, often exhibit significantly higher overall functional affinity and activity due to increased avidity compared to their monovalent counterpart [3]. In this context, the objective of this study was to examine the physicochemical properties and biological activities of a tetravalent bispecific immune cell engager and demonstrate its superior activity to its bivalent counterpart. 

Previously, we reported the sequence of a bispecific killer cell engager (BiKE) that targets the CD16a activating receptor on natural killer (NK) cells and the human epidermal growth factor receptor 2 (HER2) on cancer cells [4]. This BiKE, composed of one anti-CD16 VHH (clone C1) and one anti-HER2 VHH (clone E5), is recombinantly fused via a semi-flexible human muscle aldolase (HMA) linker [4]. For simplicity, we refer to this bivalent bispecific antibody as BiKE-E5:C1. To create a tetravalent BiKE, we employed protein engineering techniques to develop BiKE-2E5:2C1, which consists of 2 anti-CD16a VHHs fused in tandem with two anti-HER2 VHHs. Both constructs were characterized for their binding affinity to target antigens using ELISA, biolayer interferometry (BLI), and flow cytometry. The ability of these constructs to activate NK cells and induce cytotoxicity against HER2+ cancer cells was evaluated using an antibody-dependent cell-mediated cytotoxicity (ADCC) assay. NK cell activation by BiKEs was further validated by measuring the release of cytokines IFN-γ and TNF-α via ELISA. Finally, we assessed the potential of both bivalent and tetravalent BiKEs to induce fratricide due to the crosslinking of two or more NK cells.

## 2. Experimental Methods

### 2.1. Cell Culture and Handling

SKOV-3 ovarian cancer cells (Cat# HTB-77), NK92 cells (Cat# CRL-2407), and laNK92 (CD16a^F176^, Cat#PTA-8837) cells were purchased from American Type Cell Culture (Manassas, VA, USA). LaNK92 cells are not currently available at ATCC but can be purchased directly from Brink Biologics through an MTA. The laNK92 cells are genetically engineered NK92 cells that constitutively express the low-affinity CD16a receptor, making them a suitable CD16+ NK cell line for evaluation of the activity of NK cell engagers using ADCC assay [5]. Peripheral blood NK cells (PB-NK; Cat# 2W-501) were purchased from Lonza Bioscience and cultured as recommended by the vendor. LaNK92 cells were cultured in α-MEM (ThermoFisher, Somerset, NJ, USA, Cat#12561056), supplemented with 12.5% FBS (ThermoFisher, 10437028), 12.5% horse serum (ThermoFisher, 16050122), 0.2 mM myo-inositol (Sigma Aldrich, Burlington, VT, USA, Cat# I7508-100G), 0.02 mM folic acid (Sigma Aldrich, F8758-5G), 100 U/mL penicillin-streptomycin (ThermoFisher, 15140122), 0.1 mM β-mercaptoethanol (ThermoFisher, 21985023), and 100 U/mL IL-2 (Peprotech, Cranbury, NJ, USA, Cat# 200-02). NK92 and LaNK92 cells were passaged every two days and seeded at a density of 0.2 million/mL in a non-treated flask. SKOV-3 cells were cultured in McCoy’5A media (ThermoFisher, 16600108) supplemented with 10% FBS and 100 U/mL penicillin-streptomycin. SKOV-3 cells were passaged every 5–7 days and seeded in a treated flask.

### 2.2. Expression and Purification of BiKEs

The bivalent BiKE-E5:C1 was expressed and purified, as we described previously [4,6]. To express the tetravalent BiKE-2E5:2C1, the gene encoding BiKE-2E5:2C1 was synthesized and cloned into a pET28b vector by Genscript (Piscataway, NJ, USA). The vector was then transformed into a SHuffle DE3 E. coli strain (New England Biolabs, Ipswich, MA, USA, Cat# C3026J) for BiKE expression. Following inoculation of overnight culture at 30 °C in LB medium (IBI Scientific, Dubuque, IA, USA, Cat# IB49020) supplemented with 50 µg/mL kanamycin (ThermoFisher, Cat# 11815024), the culture was induced at OD_600_ of 0.5 and with 0.5 mM IPTG (Teknova, Hollister, CA, USA, Cat# I3325) and continued at 25 °C for 18 h. Cells were pelleted and resuspended in lysis buffer composed of 0.5 mg/mL lysozyme (ThermoFisher, Cat# 89833), 10 µg/mL RNase A (Sigma, Cat# R4875), 5 µg/mL DNase I (Sigma, Cat# 10104159001) in pH 7.0 tris-phosphate-saline (TBS) buffer. The suspension was then incubated at room temperature for 40 min while stirring using a plate stirrer. Subsequently, it was sonicated for 5 min on ice (3 s on, 5 s off, amplitude 75%, 120 Watt, 20 kHz). Following centrifugation at 30,000× *g* at 4 °C for 45 min, the supernatant was recovered and incubated with 1 mL slurry of Ni-NTA beads (Qiagen, Germantown, TN, USA, Cat# 30210) pre-equilibrated with 10 mL of pH 7.0 TBS, at 4 °C for 1 h. The mixture was washed once with 20 mL pH 7.0 TBS buffer containing 0.05% Tween 20 (TBS-0.05%T) and once with 20 mL DPBS (ThermoFisher, Cat# 14190144). Then, 1 mL of DPBS containing 100 U WELQut protease (ThermoFisher, Cat# EO0861) was added for overnight digestion to remove the histag. Next, BiKE-2E5:2C1 underwent a second round of purification using resin conjugated with an anti-myc tag antibody (ThermoFisher, Cat# 20169) using the purification method recommended by the manufacturer. Elution was accomplished using 1 mL of 3M sodium thiocyanate (Spectrum Chemical MFG, New Brunswick, TN, USA, Cat# S1495). Eluted BiKE was concentrated and exchanged into DPBS buffer using a Centriprep^®^30K centrifugal filter column (Sigma). The purity was assessed using SDS-PAGE gel stained with colloidal Coomassie blue (Biorad, San Francisco, CA, USA, Cat# 1610803), and the concentration was determined using the BCA assay following the manufacturer’s protocol (ThermoFisher, Cat# 23225).

### 2.3. In Silico Modeling of Bivalent BiKE-E5:C1 and Tetravalent BiKE-2E5:2C1

The amino acid sequences of BiKEs were utilized as input for structure prediction. Specifically, ChimeraX software (Version 1.8) was employed, leveraging the AlphaFold algorithm for 3D structure prediction. The ImmunoGenetics (IMGT)/V-Quest database was used to identify the complementarity-determining regions (CDRs) in each binder. Subsequently, the predicted 3D models were refined using GalaxyRefine (http://galaxy.seoklab.org/cgi-bin/submit.cgi?type=REFINE) (Access date: 8 January 2024), which performs atomic-level energy minimization and optimizes hydrogen bond networks. Finally, the quality of the refined tertiary structures was validated through Ramachandran plot analysis using PROCHECK (SAVES) (https://saves.mbi.ucla.edu/) (Access date: 8 January 2024).

### 2.4. Evaluation of the Ability of BiKE to Bind to CD16a and HER2 Antigens Using ELISA

To assess the binding activity, 0.1 µg of recombinant human CD16a (AcroBiosystem, Newark, NJ, USA, Cat# CDA-H82E8) or HER2 (AcroBiosystem, HE2-H82E2) protein in coating buffer (Biorad, Cat# BUF030C) was applied onto a 96-well plate and incubated at 4 °C overnight. After one wash with 250 µL DPBS containing 0.05% Tween 20 (DPBS-T), the plate was blocked with 3% skim milk (Merck, Cat# 1.15363.0500) in DPBS for 1.5 h. Next, 100 µL of BiKEs at equimolar concentrations, i.e., 1 µg/mL of BiKE-E5:C1 and 2 µg/mL of BiKE-2E5:2C1, was added to each well. 3G8 (anti-CD16a antibody, Invitrogen, MA1-10112) and trastuzumab (anti-HER2 antibody, Novus, NBP2-75895) were used as controls. Following five washes, 100 µL of secondary HRP-conjugated anti-myc tag antibody (Abcam, Waltham, MA, USA, Cat# ab1326), or HRP-conjugated goat anti-mouse IgG antibody (Abcam, Waltham, MA, USA, Ab6789), or HRP-conjugated goat anti-human IgG antibody (Abcam, Waltham, MA, USA, Ab97165) diluted to 0.1 ug/mL was added. Each well was then washed five times before the addition of TMB substrate (ThermoFisher, Cat# 34029). The reaction was stopped after 2–5 min with 1 N H_2_SO_4_, and measurements were taken using a Tecan Infinity^®^ PRO plate reader ((Tecan, Morrisville, NC, USA) at 450 nm with a reference wavelength of 650 nm.

### 2.5. Evaluation of the Ability of BiKE to Recognize HER2 and CD16a Antigens on Cells Using Flow Cytometry

To analyze binding activity to HER2 and CD16a, 0.5 million SKOV-3 cells (HER2+) were resuspended in 100 µL of staining buffer (DPBS containing 3% FBS). Equimolar amounts of BiKE-E5:C1 (0.5 µg) or BiKE-2E5:2C1 (1 µg) were added to the resuspended cells and incubated for 45 min at 4 °C. After incubation, the cells were washed and stained with secondary anti-myc tag antibody (Abcam, Cat#ab223896). Following three additional washes, the cells were analyzed using a flow cytometer.

Similarly, for laNK92 cells (CD16+), 0.5 million cells were resuspended in 100 µL of staining buffer and stained with either BiKE-E5:C1 (0.5 µg) or BiKE-2E5:2C1 (1 µg). After washing, cells were stained with 1 µg of rabbit anti-VHH antibody (Genscript, Piscataway, NJ, USA, Cat# A01861). The cells were then washed again and stained with goat anti-rabbit antibody conjugated with Alexa Fluor 647 (Abcam, Cat# ab150079). The stained cells were analyzed using a CytoFlex^®^ flow cytometer (Beckman Coulter, Jersey City, NJ, USA) at the Flow Cytometry Core Facility of Rutgers University. 

### 2.6. Measurement of the Binding Affinities of BiKEs Using Biolayer Interferometer 

Using a previously published method [4], the binding affinity (K_D_), rate constant of association (k_on_), and rate constant of dissociation (k_off_) were measured using a biolayer interferometer (Octet RED96e, Sartorius, Bohemia, NY, USA) at the Biophysics Core Facility at Princeton University. In brief, streptavidin biosensors (Octet^®^) were soaked inside DPBS containing 0.1% casein, with either biotinylated HER2 or biotinylated CD16a, until a 1 nm shift was noted by the instrument. Subsequently, the sensor was kept in a wash buffer for baselining, followed by loading HER2 or CD16a at concentrations of 60, 30, 15, 7.5, 3.75, 1.875, and 0 nM in the association step for 5 min. For the acquisition of the data, in the dissociation step, the sensors were dipped in the wash buffer (DPBS containing 0.1% Casein). The data were analyzed with Octet Data Analysis HT 11.1 software at the core facility. For fitting the 1:1 binding model in analysis, the sensorgrams were subtracted from the reference. The program “association and dissociation” was used to analyze affinity and kinetics.

### 2.7. Measurement of Antibody-Dependent Cellular Cytotoxicity 

For the antibody-dependent cellular cytotoxicity (ADCC) assay, we adhered to our previously published methodology [7]. Briefly, SKOV-3 cells were seeded in a 96-well plate at a density of 10,000 cells per well one day before the experiment. On the following day, the supernatants were removed, and the antibodies, diluted to the indicated concentrations, were added to each well containing SKOV-3 cells for opsonization for 30 min. Concurrently, laNK92 cells were resuspended at the specified densities to achieve different effector:target (E:T) ratios and then incubated with the SKOV-3 cells. After the incubation period, the plate was washed three times with DPBS, followed by the addition of the AlamarBlue™ (ThermoFisher, Cat#A50101) viability assay reagent and further incubation for 2 h at 37 °C. Measurements were taken using an excitation wavelength of 560 nm and an emission wavelength of 590 nm, with a bandwidth of 5.0 nm. 

### 2.8. Residual Antibody-Dependent Cellular Cytotoxicity Assay

For the residual antibody-dependent cellular cytotoxicity assay, we followed procedures adapted from a previously published method [8]. Briefly, SKOV-3 cells were seeded in a 96-well plate at a density of 10,000 cells per well one day before the experiment. On the day of the experiment, laNK92 cells were resuspended in media supplemented with BiKEs at the indicated concentrations and incubated at 37 °C for 30 min for opsonization. The laNK92 cells were then washed twice by laNK92 media at 37 °C to remove any unbound BiKEs. Subsequently, the laNK92 cells were resuspended in media for an additional 30 min to allow for the dissociation of the BiKEs. The laNK92 cells were pelleted, resuspended in media, and added to the 96-well plate containing the pre-seeded SKOV-3 cells at different E:T ratios. The plate was then incubated for 4 h at 37 °C. Following the incubation, AlamarBlue™ reagent was added, and cell viability was measured as previously described.

### 2.9. Measurement of Fratricide

To study fratricide, 50,000 laNK92 cells were first resuspended in fresh media and then added to a flat-bottom low-attachment 96-well plate (Corning, NY, USA, Cat#3370). Daratumumab (ThermoFisher, Cat# MA5-41886), 3G8 (ThermoFisher, Cat# 16-0166-65), trastuzumab (Novus, Centennial, CO, USA, Cat# NBP2-75895), BiKE-E5-C1, or BiKE-2E5:2C1 were added to each well at specified concentrations and incubated for 2 or 24 h at 37 °C. In experiments that lasted 24 h, after an initial 2 h incubation with the antibody, 50 µL of 40% AlamarBlue™ reagent diluted in NK cell culture media was added to each well, followed by an additional 22 h incubation (total 24 h). NK cell viability was measured using the AlamarBlue™ kit and protocol. 

To quantify fratricide after a 2 h incubation with antibodies, we employed the Trypan blue assay as cell viability assays that rely on the metabolic activity of the cells were not suitable. The reason is that NK cells have low metabolic activity, and incubation of NK cells with reagents such as AlamarBlue™ does not provide sufficient sensitivity (change in color) to detect dead cells. In the Trypan blue assay, 50,000 NK cells were first incubated with either daratumumab alone for 2 h or with Fc blocker or 3G8 for 30 min, followed by incubation with daratumumab at indicated concentrations for an additional 1.5 h. Aggregated cells were then dispersed into single cells by pipetting, diluted in trypan blue (0.4%, ThermoFisher Cat# 15250-061) in a 1:1 ratio, loaded onto cell counter slides, and analyzed using a Countless 3 cell counter (ThermoFisher) (n = 12). The gating method for viability was as follows: living cells (size = 70; brightness = 255; circularity = 100); dead cells (size = 0; brightness = 0; circularity = 0). A 100% viability corresponded to the total number of viable cells in the control group (treated with culture media), and relative viability was determined by the ratio of the total number of living cells in the treatment groups compared to that of the control groups.

### 2.10. Measurement of the NK Cell Degranulation and Release of Cytokines and Effector Proteins

Measurement of the surfaced CD107a (LAMP-1) on NK cells was adapted from a previously published method [4]. Briefly, 10 nM of anti-CD107a antibody was mixed with 40,000 laNK92 cells per well in a non-treated 96-well plate and incubated for two hours at 37 °C. Following incubation, the plate was centrifuged, and the cells were washed three times with staining buffer (DPBS supplemented with 3% FBS). Subsequently, the cells were stained with APC-conjugated anti-CD107a antibody (R&D Systems, Minneapolis, MN, USA, Cat#IC4800A), supplemented with Fc blocker solution (ThermoFisher, Cat#14-9161-73), and incubated for 1 h at 4 °C. Finally, the cells were centrifuged and washed three times, resuspended, and analyzed using a Beckman Coulter CytoFLEX Cytometer at the Rutgers University Flow Cytometry Core Facility.

Cytokine concentrations before and after NK cell activation were measured using our previously published method [4]. In brief, SKOV-3 cells were seeded at a density of 10,000 cells per well in a 96-well plate one day prior to the experiment. The next day, 10 nM of BiKE or control antibodies were added to the plate and incubated at 37 °C for 30 min. Then, NK cells were added to the plate and incubated for the specified duration. The plate was centrifuged at 2000× *g* for 10 min to pellet the cells, and the supernatant was transferred to a non-treated 96-well plate. The concentrations of TNF-α (R&D Systems, Cat# DY210-05), IFN-γ (R&D Systems, Cat# DY285B-05), and granzyme B (R&D Systems, Cat# DGZB00) were measured using ELISA kits following the manufacturers’ protocols.

## 3. Results and Discussion

### 3.1. Prediction of 3D Molecular Structure of BiKE-2E5:2C1

**Figure 1:**
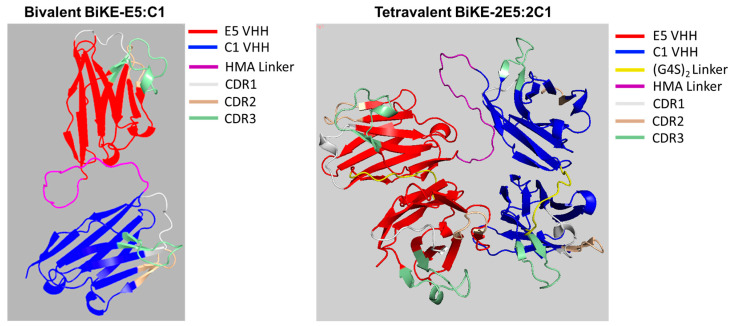
The predicted model of tetravalent and bivalent BiKEs’ tertiary structures using AlphaFold software. While the bivalent BiKE appears to have assumed a linear structure, the tetravalent BiKE appears U-shaped.

To predict the tertiary structure of the tetravalent BiKE-2E5:2C1, we utilized the AlphaFold 2 algorithm. The AlphaFold 2 is a high-precision software that uses artificial intelligence algorithms to model protein structures [9]. The predicted structures for BiKEs reveal that both antibodies are composed of nine anti-parallel β-sheets, which are typical of immunoglobulin structures (Figure 1). The structure of tetravalent BiKE-2E5:2C1 resembles a horseshoe, with all the CDRs accessible for binding, oriented toward the surface. The CDR3, shown in green, is the most variable and the longest CDR, playing a critical role in antigen binding, and is also accessible in all four binders. One advantage of the horseshoe structure, compared to a linear one, is that it creates a tighter immunological synapse, which contributes to enhanced cytotoxicity [10]. The Ramachandran analysis of both structures revealed that over 96% of the amino acid residues were in the most favored region, and less than 6% were in the allowed region, with an ERRAT score of 100%, indicating no errors in the modeled residues. 

### 3.2. Tetravalent BiKE-2E5:2C1 Binds to Its Target Antigens Similar to Bivalent BiKE-E5:C1 

**Figure 2:**
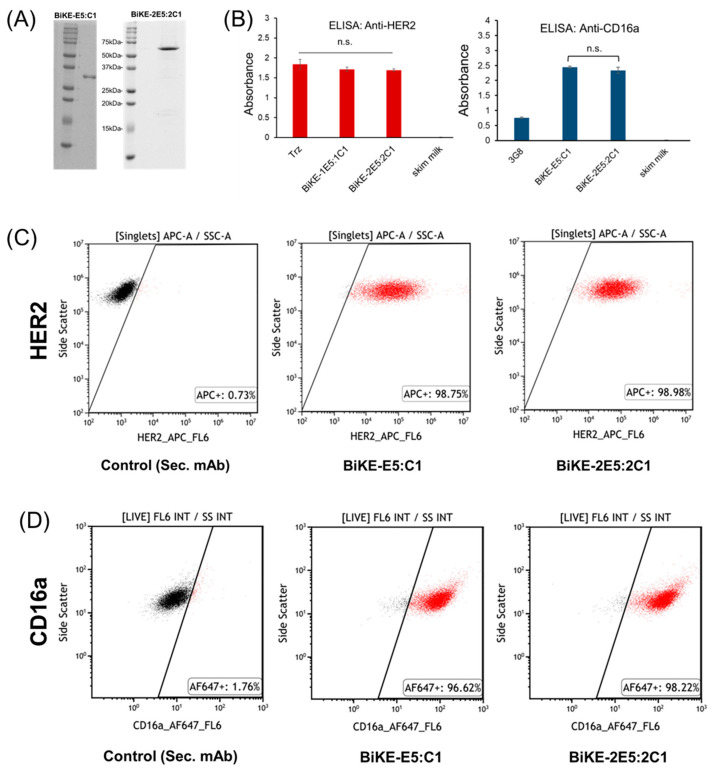
Characterization of the tetravalent BiKE-2E5:2C1 and its comparison to the bivalent BiKE-E5:C1. (**A**) SDS-PAGE analysis of the purified BiKEs. (**B**) ELISA for recognition of HER2 and CD16a antigens by the tetravalent and the bivalent BiKEs. 3G8 and trastuzumab (Trz) are used as positive controls. n.s.: not significant (*t*-test, *p* > 0.05). (**C**) Flow cytometry dot plots showing recognition of HER2 antigen on the surface of HER2+ SKOV-3 cells. (**D**) Flow cytometry dot plots showing recognition of CD16a antigen on the surface of CD16a+ laNK92 cells.

In the next step, we investigated whether the tetravalent BiKE retained its activity and could recognize its target antigens, addressing a common concern with multi-domain fusion proteins where one domain might interfere with another, rendering the fusion protein inactive. To test this, we first expressed and purified both BiKE constructs to high purity. The SDS-PAGE analysis confirmed that both BiKEs were over 95% pure, making them suitable for binding activity studies (Figure 2A). We then performed an ELISA to assess the ability of tetravalent BiKE to bind to HER2 and CD16a antigens, using the bivalent BiKE as a positive control due to its previously validated activity [4]. The ELISA results demonstrated that BiKE-2E5:2C1 could recognize both HER2 and CD16a antigens similarly to BiKE-E5:C1 (Figure 2B).

Next, we evaluated the ability of the tetravalent BiKE-2E5:2C1 to recognize HER2 and CD16a antigens on the surface of SKOV-3 (HER2+) and laNK92 (CD16a+) cells. Flow cytometry, with BiKE-E5:C1 as the positive control, showed that the tetravalent BiKE-2E5:2C1 was as effective as the bivalent BiKE-E5:C1 in binding to its target antigens on the cell surface. Overall, the ELISA and flow cytometry data indicated that both constructs were equally active, with no significant differences in antigen targeting, thereby validating that the paratopes on the tetravalent BiKE-2E5:2C1 were effectively exposed.

### 3.3. Tetravalent BiKE-2E5:2C1 Exhibits Picomolar Affinity toward Its Target Antigens 

**Figure 3:**
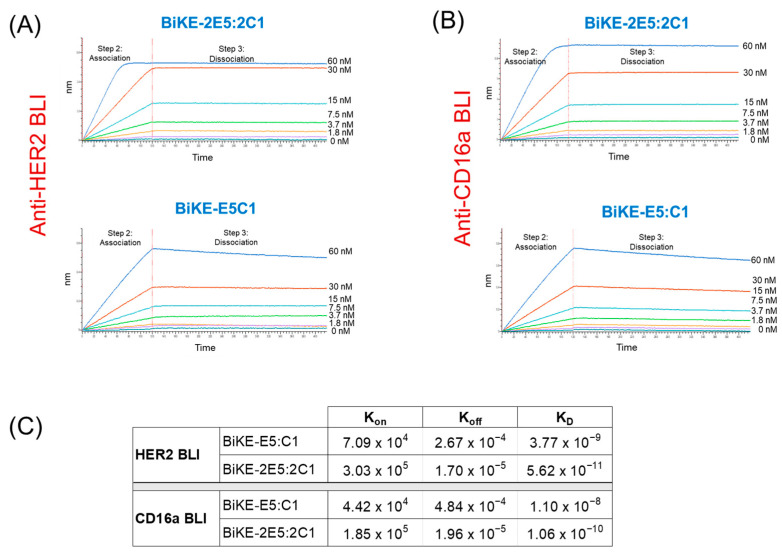
Measurement of K_D_ (binding constant), K_on_ (association constant), and K_off_ (dissociation constant) of the tetravalent BiKE-2E5:2C1 and the bivalent BiKE:E5C1 using BLI. (**A**,**B**) BLI sensograms showing the binding kinetics of BiKEs for HER2 and CD16a antigens. (**C**) The measured K_D,_ K_off_, and K_on_ values of BiKEs for HER2 and CD16a antigens.

We have previously demonstrated that bivalent BiKE-E5:C1, due to its high-affinity interaction with the CD16a receptor, could activate the NK cells to kill cancer cells more efficiently than the best-in-class anti-HER2 antibody, trastuzumab [4]. Given the importance of antibody affinity toward their target antigens in determining their activity, we investigated whether tetravalent BiKE-2E5:2C1 could bind to its target antigens with higher avidity than bivalent BiKE-E5C1. To this end, we employed BLI to measure the affinity of both BiKEs toward the HER2 antigen. The BLI sensograms revealed K_D_ values of 56 pM for tetravalent BiKE-2E5:2C1 and 3.8 nM for the bivalent BiKE-E5:C1 (Figure 3A–C). Next, we assessed the affinities of BiKEs toward the CD16a antigen. The BLI sensograms showed the affinities of 106 pM for the tetravalent BiKE-2E5:2C1 and 11 nM for bivalent BiKE-E5:C1 (Figure 3B,C). While most antibodies have K_D_ values in the range of 10^−7^ to 10^−9^ M, high-affinity antibodies have K_D_ values in the sub-nanomolar range (10^−9^ M). Therefore, the tetravalent BiKE-2E5:2C1 can be considered a very high-affinity antibody due to its picomolar affinity toward its target antigens. Overall, the BLI data demonstrated a significant enhancement of binding affinity when the bivalent BiKE-E5:C1 was engineered as the tetravalent BiKE-2E5:2C1, conferring enhanced affinity through avidity. These findings prompted us to conduct an ADCC assay to measure and compare the anticancer activity of the tetravalent and the bivalent BiKEs.

### 3.4. Tetravalent BiKE-2E5:2C1 Has Higher Anticancer Activity than Bivalent BiKE-E5:C1

**Figure 4:**
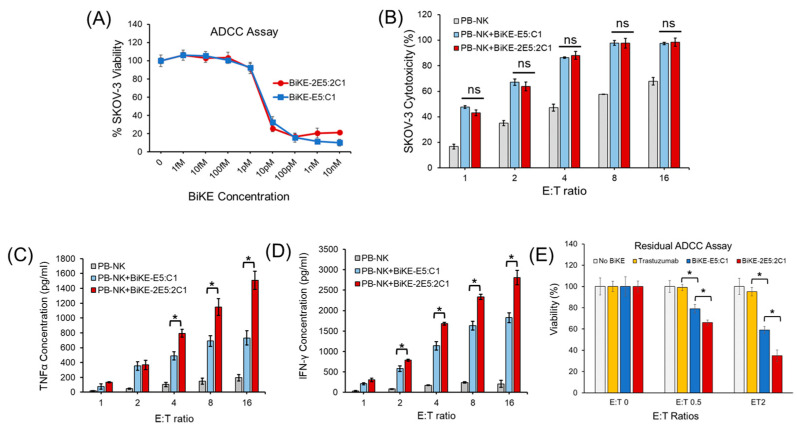
Measurement of the difference in anticancer activity of the tetravalent BiKE-2E5:2C1 and the bivalent BiKE-E5:C1. (**A**) Classical ADCC assay using laNK92 cells and SKOV-3 cells at fixed E:T ratio of 4 but variable BiKE concentrations. (**B**) Classical ADCC assay using PB-NK cells and SKOV-3 cells at various E:T ratios but fixed BiKE concentration (100 nM). (**C**,**D**) Measurement of the IFN-γ and TNF-α after incubation of SKOV-3 cells with PB-NK cells in presence of BiKE using ELISA. (**E**) Residual ADCC assay using laNK92 cells and SKOV-3 cells at different E:T ratios. * *t*-test, *p* < 0.05, ns: not significant (n = 3).

Multivalent antibodies contain multiple paratopes, allowing them to target selected antigens of interest with enhanced avidity and reduced dissociation rates, ultimately resulting in higher efficacy. Clinical data have shown a direct correlation between the affinity of antibodies for CD16a receptors and therapeutic outcomes [11,12]. Given that the tetravalent BiKE-2E5:2C1 exhibited approximately 100-fold higher affinity toward its target antigens compared to the bivalent BiKE-E5:C1, we sought to determine if this increased affinity would translate into enhanced anticancer activity. To test this, we first conducted a classical ADCC assay using laNK92 (CD16a+) cells as effector cells and SKOV-3 cells as targets. We have previously demonstrated that the bivalent BiKE could activate CD16-expressing immune cells, including PB-NK cells, laNK92 cells, and THP-1-CD16a macrophages [7]. For the ADCC assay in this study, we utilized both laNK92 and PB-NK cells. The results indicated no significant difference in anticancer activity between the tetravalent and bivalent BiKEs regardless of the NK cell type used (Figure 4A,B). This observation was unexpected because measurement of the concentrations of released cytokines (i.e., TNF-α and IFN-γ) from the PB-NK cells illustrated that at almost all E:T ratios, the tetravalent BiKE-2E5:2C1 activated NK cells more efficiently than the bivalent BiKE-E5:C1 (*t*-test, *p* < 0.05) (Figure 4C,D). To resolve this ambiguity, we performed a residual ADCC assay, which serves as a proxy for the in vivo persistence of an antibody. We removed excess unbound BiKE in each well and placed BiKE-coated NK cells under sink conditions to accelerate the dissociation of lower affinity BiKE molecules. This approach allowed high affinity BiKE molecules that remained bound to the surface of the NK cells to interact with SKOV-3 cancer cells. The rationale was that the higher avidity and the resultant lower k_off_ for the CD16a receptor of the tetravalent BiKE would lead to greater antibody retention on the pre-opsonized NK cells. The results of the residual ADCC assay demonstrated that the tetravalent BiKE-2E5:2C1 was significantly more effective in killing cancer cells than the bivalent BiKE-E5:C1 (Figure 4E). The prior literature on potent high-affinity antibodies targeting CD16a receptors also supports the fact that residual ADCC is a more effective method for capturing the differences between tetravalent and bivalent antibodies, especially when both are highly potent [8]. Our earlier work has shown that the bivalent BiKE could activate NK cells to kill not only HER2-positive cancer cells but also those with low HER2 expression [7]. Given that the tetravalent BiKE exhibits higher affinity with lower K_off_ for both CD16a and HER2 antigens and is more efficient at killing cancer cells under sink conditions than its bivalent counterpart, it may offer the potential to target and eradicate low HER2-expressing cancer cells with greater specificity and efficiency. This could ultimately lead to improved therapeutic responses with fewer side effects in low HER2-expressing cancer patients (e.g., breast, ovarian, and lung) due to prolonged recruitment and activation of immune effector cells.

### 3.5. Tetravalent BiKE-2E5:2C1 Targeting CD16a Receptors Does Not Mediate NK Fratricide

In this study, we evaluated the safety of our BiKE platform by assessing the risk of intercellular CD16a-CD16a crosslinking and the mediation of NK fratricide—a phenomenon where NK cells kill each other, often seen as an unwanted side effect. Fratricide has been documented with daratumumab, which is a bivalent anti-CD38 antibody [13,14]. Clinical data indicate that treatment with daratumumab leads to the rapid depletion of approximately 85% of NK cells, lasting up to six months post-treatment discontinuation [15,16]. 

**Figure 5:**
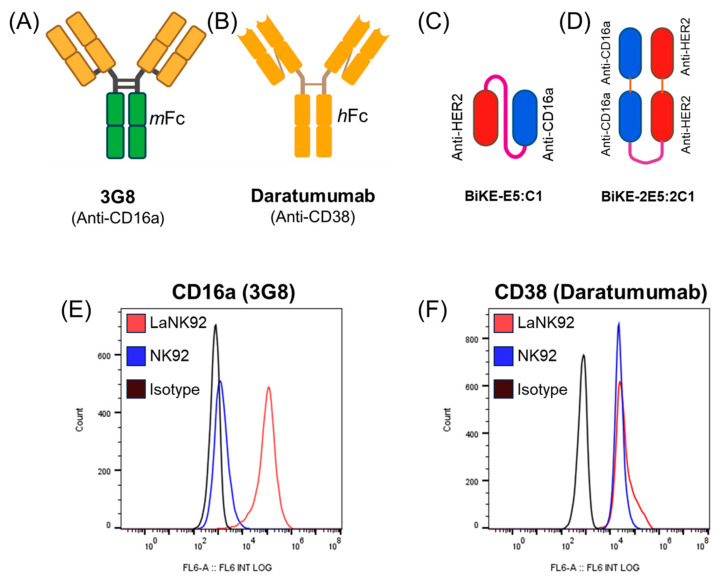
Specifications of the antibodies and cells used in examining fratricide. (**A**) Bivalent anti-CD16a antibody (3G8) with mouse IgG Fc domain. (**B**) Bivalent anti-CD38 antibody (daratumumab) with human IgG Fc domain. (**C**,**D**) schematic representation of tetravalent and bivalent BiKEs. (**E**) flow cytometry histogram demonstrating the expression of CD16a receptor on laNK92 cells but not NK92 cells using 3G8 antibody. (**F**) flow cytometry histogram showing expression of CD38 receptors on both laNK92 and NK92 cells using daratumumab.

Given that BiKE-2E5:2C1 is a tetravalent antibody capable of crosslinking NK cells via CD16a activating receptors, we investigated whether it could mediate NK cell fratricide. For antibody controls, we utilized daratumumab, an anti-CD38 antibody with a human IgG Fc domain, and 3G8, an anti-CD16a antibody with a mouse IgG Fc domain (Figure 5A,B). The mouse Fc domain of the 3G8 antibody is unable to engage CD16a receptors on human NK cells, thereby restricting its interaction with NK cells solely through the Fab regions. Bivalent BiKE-E5:C1, which is incapable of crosslinking CD16a receptors, was used as a negative BiKE control. For clarity, we illustrated the molecular structures of the tetravalent and bivalent BiKEs using a ball-and-stick model (Figure 5C,D). As cell controls, we employed NK92 cells (CD16−, CD38+) and laNK92 cells (CD16+, CD38+) [17]. While NK-92 cells cannot mediate ADCC [18], laNK92 cells are highly efficient in mediating ADCC [4,7]. Therefore, the NK92/laNK92 pair were selected as effector cells for the fratricide studies. 

Initially, NK92 cells, laNK92 cells, daratumumab, and 3G8 were used to validate the expression of CD38 receptors on both cell lines, as well as the presence of CD16a receptors on laNK92 cells but not NK92 cells (Figure 5E,F). While the ability of laNK92 cells to induce ADCC has previously been demonstrated by our group [4], the inability of NK92 cells to induce ADCC, due to the lack of CD16a expression, was confirmed using SKOV-3 cells (HER2+) as target cells (Supplementary Figure S1). 

**Figure 6:**
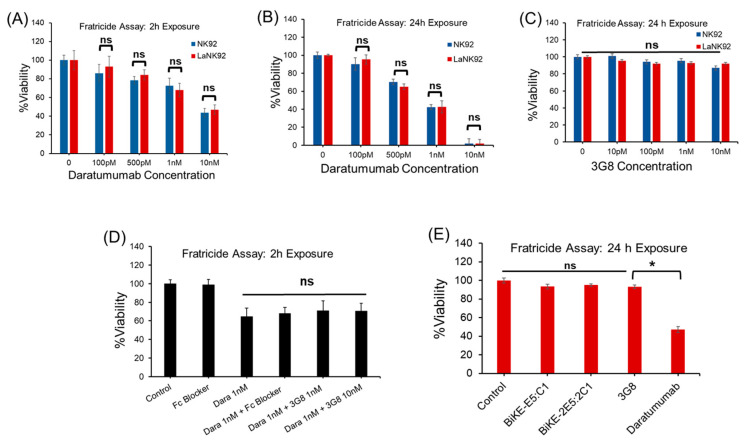
Evaluation of the ability of tetravalent BiKE to induce fratricide by measuring the cell viability after incubation of NK cells with antibodies. (**A**,**B**) Fratricide (ADCC) assay using NK92 and laNK92 cells in combination with daratumumab at different concentrations. (**C**) Fratricide assay using NK92 and laNK92 cells in combination with 3G8 antibody at different concentrations. (**D**) Fratricide assay using daratumumab in combination with 3G8 or Fc blocker at different concentrations. (**E**) Fratricide assay using laNK92 cells and tetravalent and bivalent BiKEs along with control antibodies. * *t*-test, *p* < 0.05. ns: not significant.

Using the aforementioned cells and antibodies, we conducted fratricide assays by incubating NK92 and laNK92 cells with daratumumab, 3G8, or a combination of daratumumab and 38 antibodies. The objective was to determine whether fratricide occurs due to CD38-CD38 crosslinking or if it could be initiated through CD38-CD16a or CD16a-CD16a crosslinking. To test this hypothesis, we performed three independent fratricide assays. The results revealed that daratumumab was equally toxic to both NK92 and laNK92 cells regardless of incubation time, whereas the 3G8 antibody did not mediate fratricide (Figure 6A–C). These observations indicated that daratumumab’s toxicity was due to the crosslinking of CD38-CD38 and not CD16a-C38 crosslinking, as NK92 cells mediated fratricide similar to laNK92 cells. This suggests that CD38-CD38 crosslinking, rather than CD16a-CD16a, is the major player in fratricide. To validate this observation, we incubated laNK92 cells with daratumumab alone or with daratumumab plus 3G8 antibodies. The results revealed that blocking of CD16a with the 3G8 antibody or Fc blocker did not significantly impact daratumumab’s fratricide effect, ruling out CD16a-CD38 crosslinking as the culprit (Figure 6D) (Supplementary Figure S2). Overall, our data indicate that daratumumab is an agonistic CD38 antibody that mediates fratricide through NK cells’ natural cytotoxicity capability, which is the innate ability of NK cells to lyse tumor or virally infected target cells. While some reports suggest that daratumumab mediates fratricide through crosslinking of CD16a-CD38 receptors, which is an antibody-dependent mechanism (ADCC) [13], our data do not support this idea and are more in agreement with early report by Sconocchia et al. [19]. 

Finally, we examined whether the tetravalent BiKE-2E5:2C1 could mediate fratricide. The results of the fratricide assay showed that similar to the 3G8 antibody, the tetravalent BiKE-2E5:2C1 did not mediate fratricide (Figure 6E). 

**Figure 7:**
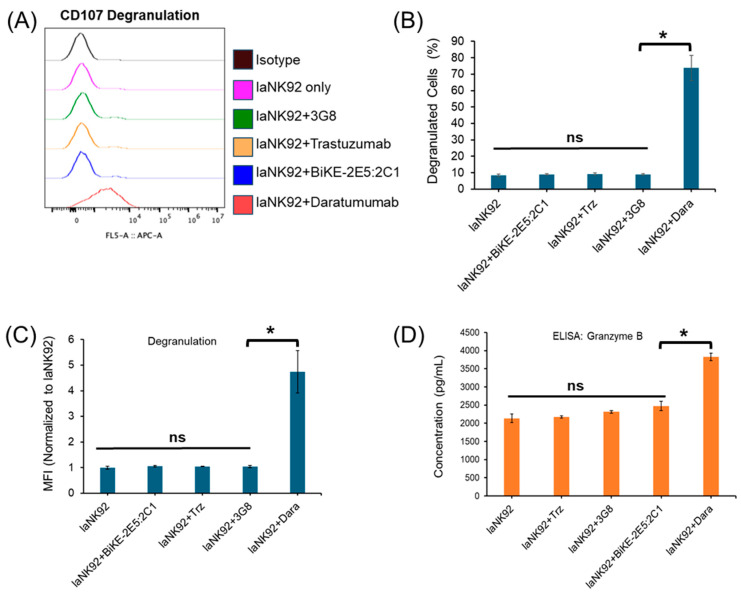
Determination of NK cell activation. (**A**–**C**) Measurement of the degranulation of laNK92 cells after incubation with tetravalent BiKE, trastuzumab (Trz.), 3G8, and daratumumab at 10 nM concentration (2 h exposure time) using flow cytometry. (**D**) Measurement of the concentration of granzyme B from antibody treated laNK92 cells using ELISA. * *t*-test, *p* < 0.05. ns: not significant.

To elucidate the mechanism of action, we measured the degranulation of laNK92 cells and the release of granzyme B from activated NK cells. Flow cytometry histograms and ELISA results illustrated that only daratumumab was capable of degranulating NK cells, resulting in the release of granzyme B (Figure 7A–C) (Supplementary Figure S3). Given that CD38, through its enzymatic functions, can regulate granule polarization and degranulation of the NK cells [20,21], this explains daratumumab’s ability to activate NK cells, leading to fratricide. Overall, unlike daratumumab, the tetravalent BiKE-2E5:2C1 did not compromise NK cell viability, indicating its potential to serve as a safe therapeutic platform.

## 4. Conclusions

The data presented in this manuscript demonstrate that a tetravalent BiKE can be engineered from its bivalent counterpart by fusing VHH units in tandem. The resulting tetravalent BiKE exhibits high affinity toward its target antigens due to avidity. This study is the first in the literature to show the successful engineering of a tetravalent immune cell engager composed of tandem VHH units, which achieves high affinity and anticancer activity without mediating fratricide.

## Data Availability

All data relevant to this study are included in the article or uploaded as Supplementary Information. The original contributions presented in this study are included in the article/Supplementary Materials; further inquiries can be directed to the corresponding author.

## Supplementary Materials

The following supporting information can be downloaded at https://www.mdpi.com/article/10.3390/antib13030075/s1, Figure S1: ADCC assay using NK92 as effector cells and SKOV-3 as target cells at E:T ratio of 4. BiKE-E5:C1 and trastuzumab (Trz) were used at 10 nM concentration. n.s.: not significant, *t*-test *p* > 0.05.; Figure S2: Fratricide assay using daratumumab alone or in combination with 3G8 or Fc blocker at different concentrations (24 h incubation time); Figure S3: Flow cytometry data for degranulation of laNK92 cells after incubation with antibodies.
