# Supplementary material for: A Novel Tetravalent Bispecific Immune Cell Engager Activates Natural Killer Cells to Kill Cancer Cells without Mediating Fratricide
Source: Antibodies (Basel). 2024 Sep 10;13(3):75. doi: 10.3390/antib13030075 (PMC11417942; doi:10.3390/antib13030075)
Supplement: Supplementary file 1 [file antibodies-13-00075-s001.zip › antibodies-3121464-supplementary.pdf]

Supplementary Materials

A Novel Tetravalent Bispecific Immune Cell Engager Activates Natural Killer Cells to Kill Cancer Cells Without Mediating Fratricide

+Ge Yang[1], +Shahryar Khoshtinat Nikkhoi[1], Hajar Owji[1], Geng Li[1], Mohammad Massumi[1], Jessica Cervelli[2], Venu Gopal Vandavasi[3], and *Arash Hatefi[1,4]

[1]Department of Pharmaceutics, Rutgers University, Piscataway, NJ 08854, USA

[2]Environmental and Occupational Health Science Institute, Flow Cytometry Core Facility, Rutgers University, Piscataway NJ 08854, USA

[3]Department of Chemistry, Biophysics Core Facility, Princeton University, Princeton, NJ, 08544, USA

[4]Cancer Pharmacology Program, Cancer Institute of New Jersey, New Brunswick, NJ 08901, USA

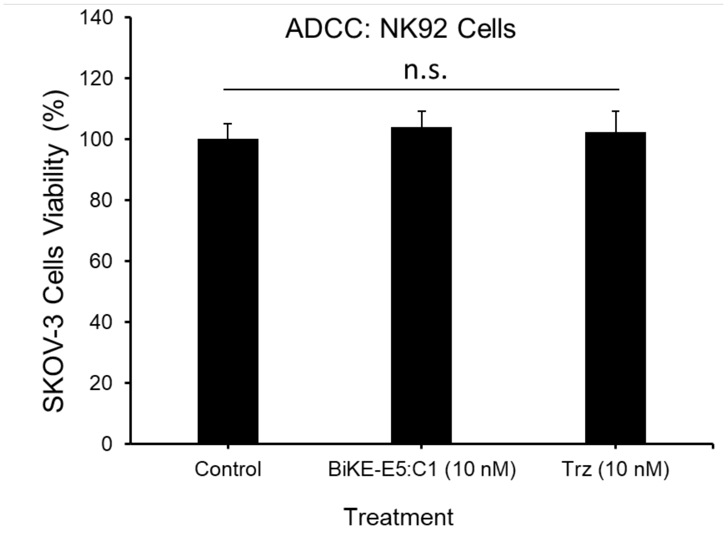

Figure S1: ADCC assay using NK92 as effector cells and SKOV-3 as target cells at E:T ratio of 4. BiKE-E5:C1 and trastuzumab (Trz) were used at 10 nM concentration. n.s.: not significant, t-test, $p>0.05$.

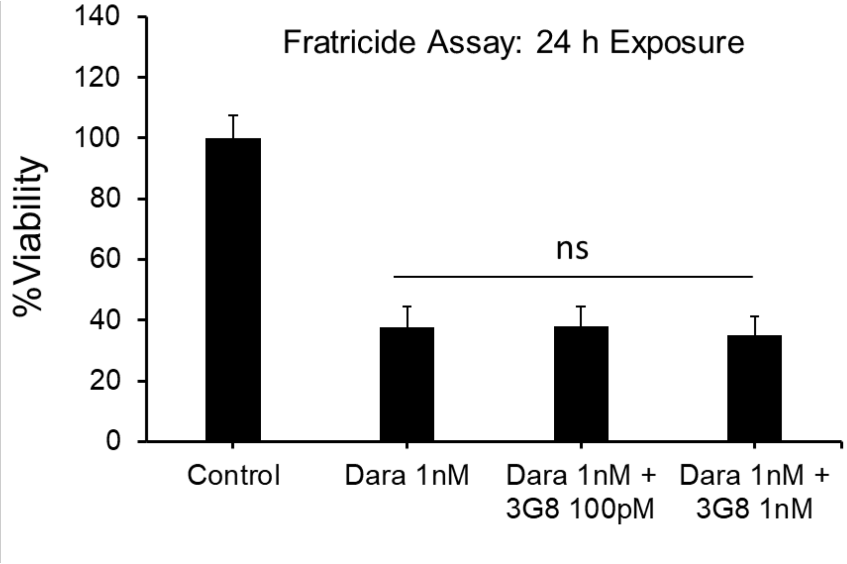

Figure S2: Fratricide assay using daratumumab alone or in combination with 3G8 or Fc blocker at different concentrations (24 h incubation time).

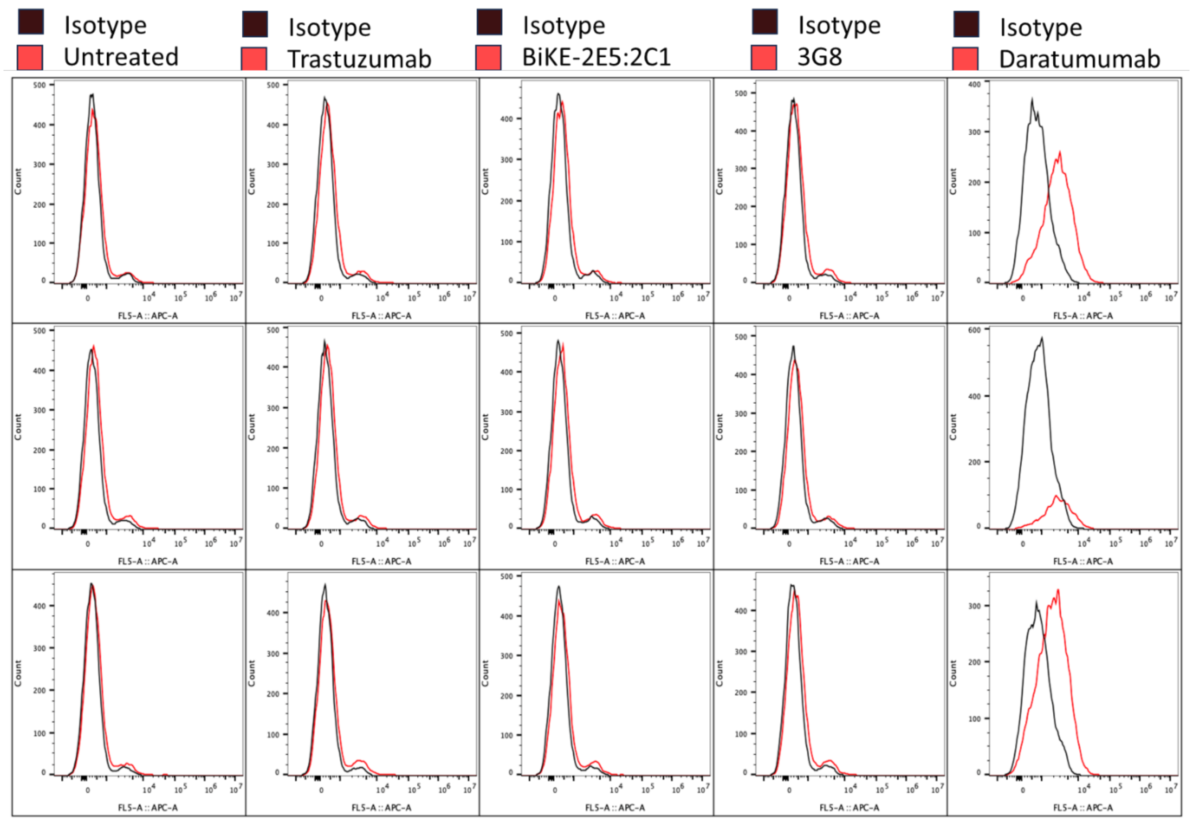

Figure S3: Flow cytometry data for degranulation of laNK92 cells after incubation with antibodies.
